# Step Climbing Control of Snake Robot with Prismatic Joints

**DOI:** 10.3390/s22134920

**Published:** 2022-06-29

**Authors:** Yuta Iguchi, Mizuki Nakajima, Ryo Ariizumi, Motoyasu Tanaka

**Affiliations:** 1 Department of Mechanical and Intelligent Systems Engineering, The University of Electro-Communications, Chofu 182-8585, Japan; master2021iguchi@rc.mce.uec.ac.jp (Y.I.); mtanaka@uec.ac.jp (M.T.); 2 Department of Mechanical Science and Engineering, Nagoya University, Nagoya 464-8601, Japan; ryo.ariizumi@mae.nagoya-u.ac.jp

**Keywords:** prismatic joints, linear joints, redundancy, snake robot, step climbing

## Abstract

The ultimate goal of this research study is to perform continuous rather than sequential movements of prismatic joints for effective motion of a snake robot with prismatic joints in a complex terrain. We present herein a control method for robotic step climbing. This method is composed of two parts: the first involves the shift reference generator that generates the joint motion for climbing a step, and the other is use of the trajectory tracking controller, which generates the joint motion for the head to track the target trajectory. In this method, prismatic joints are divided into those that are directly controlled for climbing a step and those that are represented as redundancies. By directly controlling the link length, it is possible to prevent the trailing part from back motion when climbing a step, and to avoid a singular configuration in the parts represented as redundancies. A snake robot that has rotational and prismatic joints and can move in three-dimensions was developed, and the effectiveness of the proposed method was demonstrated by experiments using this robot. In the experiment, it was confirmed that the proposed method realizes the step climbing, and the link length limitation using the sigmoid function works effectively.

## 1. Introduction

Despite their long, slender, and simple shape, biological snakes are able to move across flat surfaces and walls, and climb up and down trees. In addition, some snakes swim underwater and glide through the air. To move in different environments, snakes use different modes of locomotion. Snakes use four main land movements, namely, serpentine, sidewinding, rectilinear, and concertina [1]. Many robots have been developed to mimic the various locomotion patterns of snakes [2].

Serpentine movement is the typical locomotion of biological snakes. The snake uses the difference in frictional force between the direction of its trunk and the direction perpendicular to it to generate a propulsive force through the lateral undulation of its body. For the purpose of performing biological-like motion, a serpenoid curve [1], three-dimensional motion [2,3], central pattern generator (CPG)-based control [4,5], and decentralized control [6] have been proposed. In contrast, numerous studies have been conducted using a model-based control approach in which the controller was designed based on a mathematical model. This approach enables the autonomous generation of snake robot behaviors to achieve control objectives, and the trajectory tracking of the robot head is realized on a flat plane [7,8,9], on a step [10], and on two nonparallel planes [11]. Accordingly, control methods have been proposed for path tracking of the center of mass [12] by using a model that considers the slipping of wheels. These studies dealt only with joint rotations and did not consider link extension or shrinking at all. In the cases in which cord-like robots were controlled with extendable mechanisms, predesigned peristalsis is mainly used. Robots that mimic the peristalsis performed by earthworms [13] have been developed and researched [14,15,16,17,18]. In addition, a robot that mimics the behavior of an inchworm has been studied in [19,20]. In contrast, a predesign method that was not a biomimicry was proposed in [21]. In this method, joint rotation and link extension/shrinking were predesigned as continuous curves, and three-dimensional motion with simultaneous joint rotation and link extension/shrinking were realized.

However, when using sequence control or a predesigned method described above, only fixed extendable movements can be performed. For example, locomotion that is designed to execute along a straight path cannot be applied directly to a turning motion. Thus, an extension and shrinking motion must be designed for each assumed motion. Therefore, when the environment or motion changes, it is desirable to change adaptively the extension/shrinking of the robot’s link and the rotation of the robot’s joints according to the situation.

Additionally, a head trajectory tracking control method for a two-dimensional plane was proposed in [22] as a method to utilize adaptive link extension/shrinking and joint rotation. In this method, the degrees of freedom for link stretching and joint rotation are represented collectively as redundancies, thereby allowing the robot to adaptively stretch links and rotate joints to achieve secondary control objectives other than the head trajectory tracking. In addition, as the link length limit for stretching is taken into account, the link lengths do not exceed hardware limits. However, verification has only been conducted based on simulations, and no experiments on actual equipment have been conducted.

The objectives of this study are to extend the control method proposed in [22] to nonplanar environments, and to utilize adaptive extension and shrinking of the links in more complex environments for a snake robot with extendable links. As the first step, we assume a step composed of two parallel planes as an environment, and propose a control method for a snake robot to move in the environment. The snake robot to be controlled consists of rotational joints, prismatic joints, and passive wheels; and the lengths of its links can be varied by the prismatic joints. The robot is described as a redundant system with redundancy with respect to link extension/shrinking and joint rotation. Furthermore, redundancy can be used to achieve secondary control objectives. As a way to keep the link length within hardware limits, a sigmoidal function was introduced into the model, as described in [22]. The effectiveness of the control method was demonstrated by experiments using an actual robot.

To ascend or descend a step, the robot must move between two planes with proper rotation of joints and a proper link length. If no consideration is given, the robot’s wheels will not be in proper contact with the respective planes, and the robot will not be able to generate the force from its wheels to move forward. To maintain proper contact between the wheels and planes, the link lengths should be expressed as controlled variables and controlled directly. However, this is inconsistent with the expression and use of the extension and shrinking degrees of freedom as redundancy. Therefore, in this study, the link length is divided into two parts: one for direct control and the other for use as redundancy. The two parts are used together to solve the problem.

The contributions of this study are listed below.

- We propose a control method for climbing a step by extending and retracting a link, and by rotating a joint By dividing the degrees of freedom of the link lengths into two parts, one part controls the link length directly, and the other part uses the link length as a redundancy. The proposed control method can achieve both of the following: (i) link extension/shrinking to move between two planes to achieve step climbing, and (ii) link extension/shrinking in accordance with joint rotation.
- We have developed an actual snake robot that can extend and retract links by prismatic and rotate joints, and demonstrate the effectiveness of a control method that adaptively utilizes the extension and shrinking of degrees of freedom through experiments. This study was the first demonstration using an actual robot, as [22] only verified the method with simulations.

## 2. Model

### 2.1. Problem Setting

**Figure 1:**
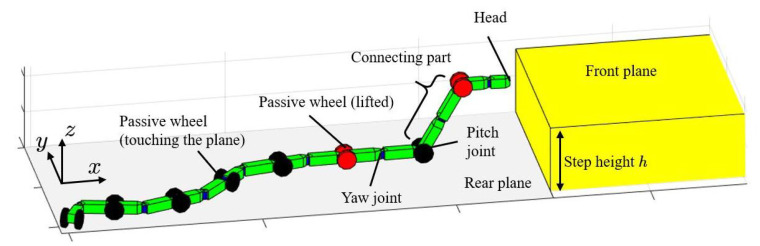
A snake robot and a step.

Figure 1 shows a snake robot which can rotate its joints and change its link length and the step environment. The robot consists of variable-length links connected alternately by yaw and pitch rotational joints. It also comprises a pair of passive wheels mounted on the same axis as the pitch joints. The link changes the length according to the motion of the prismatic joint. Passive wheels simulate friction conditions. This means that wheels are less likely to skid in a manner similar to biological snakes. Accordingly, the robot can move while undulating by rotating joints properly. The assumed environment is a step with a height equal to *h*. The plane on the step is called the front plane, and the plane that the robot initially touches is called the rear plane.

This robot can arbitrarily change the link length. In the implementation of the motion control of this robot, the use of the degrees of freedom about the link length (telescopic degrees of freedom) is an important issue. In this study, the telescopic degrees of freedom are divided into two parts for control: the part that is directly controlled for the shift of connecting part, and the part that is expressed as redundancy and used for singular posture avoidance.

In designing the step-climbing motion, we adopted the method of [10] for the snake robot without prismatic joints. This method introduces *a connecting part* in the body of the snake robot that connects the forward and rear planes. By shifting the connecting part backward at the appropriate time, the robot can move from the rear to the front plane. By setting the yaw joint angle included in the connecting part to zero, the wheels at the both end of the connecting part can be properly contacted with respect to both the forward and rear planes. The robot is represented by two models. One model is a projection onto the xy-plane, and the other is a pitch-angle model for the connecting part.

**Figure 2:**
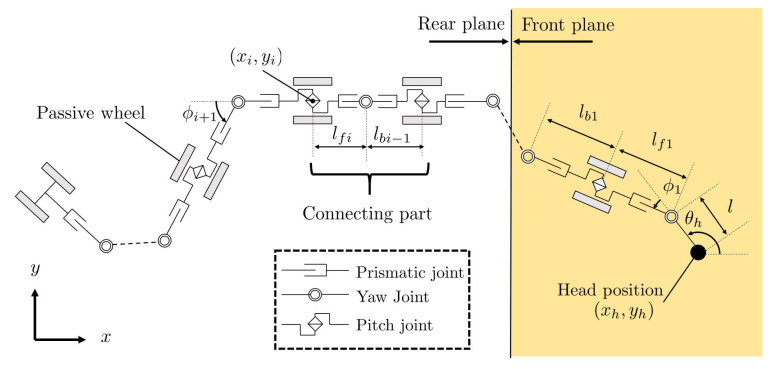
A projection model on the xy-plane.

Figure 2 shows the projection model onto the xy-plane. In this model, let $l_{fi}$ and $l_{bi}$ be the forward and backward link lengths of the *i*th pair of passive wheels, *n* be the number of pairs of passive wheels, $w={\left[x_{h},y_{h},\theta_{h}\right]}^{⊤}$ be the position and orientation of the robot’s head, $\phi_{i}$ be the *i*th yaw joint, $\psi_{i}$ be the relative angle of the *i*th pitch joint, and $\psi_{h}$ be the absolute pitch angle of the head link. Let us define $\phi ={\left[\phi_{1},\cdots ,\phi_{n}\right]}^{⊤}$, $\psi ={\left[\psi_{1},\cdots ,\psi_{n-1}\right]}^{⊤}$, $l_{f}={\left[l_{f1},\cdots ,l_{fn}\right]}^{⊤},l_{b}={\left[l_{b1},\cdots ,l_{bn-1}\right]}^{⊤}$, $l_{all}={\left[l_{f}^{⊤},\;l_{b}^{⊤}\right]}^{⊤}$, $\Psi_{i}=\psi_{h}+\sum_{j=1}^{i-1}\psi_{j}$, $|\Psi_{i}|<\frac{\pi }{2}$.

### 2.2. Kinematic Model

In the kinematic model, it is assumed that the wheels do not skid in the transverse direction. The coefficient of friction in the transverse direction of the wheel is much larger than the coefficient of friction in the longitudinal direction (rolling friction), and the input is derived to satisfy the constraints (the input that prevents the wheel from skidding) based on the control model. Therefore, wheel skidding is negligibly small. In contrast, model errors due to joint angle resolution and mechanical stiffness of the robot can cause minor skidding. Although skidding causes an error of the controlled variables from the target, it can be corrected by the feedback control. Let the *x* and *y* positions of the center of *i*th pair of wheels be $x_{i}$ and $y_{i}$, respectively, and the absolute angle of the links adjacent to the *i*th pair of wheels be $\theta_{i}$. The velocity constraint equation, which ensures that the passive wheel will not skid, is expressed as

(1) $$\begin{array}{c} \dot{x_{i}}\sin\theta_{i}-\dot{y_{i}}\cos\theta_{i}=0. \end{array}$$

A simultaneous set of Equation (1) for all pairs of passive wheels is represented as

(2) $$A\dot{w}=\left[\begin{array}{cc} B_{\phi } & B_{l} \end{array}\right]u$$

(3) $$\begin{array}{cc} u & =\left[\begin{array}{c} \dot{\phi }\\ {\dot{l}}_{all} \end{array}\right], \end{array}$$

where *u* is the control input, the *i*th row of (2) represents the velocity constraint equation for the *i*th wheel pair, and $A\in R^{n\times 3},B_{\phi }\in R^{n\times n},B_{l}\in R^{n\times (2n-1)}$. Let $B_{l\left[i:j\right]}$ be the matrix consisting of i,⋯,j columns of $B_{l}$ and $B_{\phi }$:

(4) $$B_{\phi }=\left[\begin{array}{ccccc} l_{f1} & \; & & 0\\ \; & l_{f2} & \; & & \\ \; & \; & \ddots & \\ {}* & \; & & l_{fn} \end{array}\right],$$

(5) $$\begin{array}{cc} B_{l[1:n-1]} & =B_{l[n+1:2n-1]} \end{array}$$

(6) $$\begin{array}{cc} \; & =\left[\begin{array}{ccc} 0 & \cdots & 0\\ b_{2,1} & \; & 0\\ \vdots & \ddots & \\ b_{n,1} & \cdots & b_{n,n} \end{array}\right], \end{array}$$

(7) $$\begin{array}{cc} B_{l[n]} & =0, \end{array}$$

(8) $$\begin{array}{cc} b_{\left[i,j\right]} & =-\sin\sum_{k=j+1}^{i}\phi_{k}. \end{array}$$

The yaw angle ϕ and the actual link length (not the link length of the projection model) of the robot are limited depending on hardware. To constrain the *j*th link length of the actual robot ${\bar{l}}_{j}$, $l_{j}$ is expressed using the standard sigmoidal function and the mediating variable $\gamma_{j}$. The standard sigmoidal function is expressed as

(9) $$\begin{array}{c} f\left(x\right)=\frac{1}{1+\exp(-x)}. \end{array}$$

Using the sigmoidal function (9), the *j*th actual link length of the robot ${\bar{l}}_{j}$ can be expressed as

(10) $$\begin{array}{cc} {\bar{l}}_{j} & =\left({\bar{l}}_{U}-{\bar{l}}_{L}\right)f\left(\gamma_{j}\right)+{\bar{l}}_{L}, \end{array}$$

(11) $$\begin{array}{cc} {\dot{\bar{l}}}_{j} & =\left({\bar{l}}_{U}-{\bar{l}}_{L}\right)\left\{1-f\left(\gamma_{j}\right)\right\}f\left(\gamma_{j}\right), \end{array}$$

where ${\bar{l}}_{U}\neq 0$ and ${\bar{l}}_{L}\neq 0$ are the maximum and minimum values of the link length, respectively, and ${\bar{l}}_{L}\leq {\bar{l}}_{j}\leq {\bar{l}}_{U}$. The link lengths $l_{fi},l_{bi}$ of the projection model are calculated using the absolute pitch angle $\Psi_{i}$, along with the mediating variable $\gamma_{fi},\gamma_{bi}$, as

(12) $$\begin{array}{cc} l_{fi} & =\left\{\left({\bar{l}}_{U}-{\bar{l}}_{L}\right)f\left(\gamma_{fi}\right)+{\bar{l}}_{L}\right\}\cos\Psi_{i}, \end{array}$$

(13) $$\begin{array}{cc} l_{bi} & =\left\{\left({\bar{l}}_{U}-{\bar{l}}_{L}\right)f\left(\gamma_{bi}\right)+{\bar{l}}_{L}\right\}\cos\Psi_{i+1}. \end{array}$$

When the link length is expressed by Equations (10)–(13), the actual link length is limited to ${\bar{l}}_{L}\leq {\bar{l}}_{j}\leq {\bar{l}}_{U}$. Therefore, the link length does not exceed the movable limit, regardless of the input. Assuming that $\psi_{h}={\dot{\psi }}_{h}=0$ is valid as the robot’s head continues to move on the same plane, when it moves between the two planes [10], the following equation can be used.

(14) $${\dot{l}}_{all}=\left[\begin{array}{cc} C_{1} & C_{2} \end{array}\right]\left[\begin{array}{c} {\dot{\gamma }}_{all}\\ \dot{\psi } \end{array}\right],$$

where

(15) $$\begin{array}{cc} C_{1} & =\left[\begin{array}{cccccc} g_{1}\left(\gamma_{f1}\right)\cos\Psi_{1} & \; & & \; & & 0\\ \; & \ddots & \; & & \; & \\ \; & \; & g_{1}\left(\gamma_{fn}\right)\cos\Psi_{n} & \; & & \\ \; & \; & & g_{1}\left(\gamma_{b1}\right)\cos\Psi_{2} & \; & \\ \; & \; & & \; & \ddots & \\ 0 & \; & & \; & & g_{1}\left(\gamma_{bn-1}\right)\cos\Psi_{n} \end{array}\right], \end{array}$$

(16) $$\begin{array}{cc} g_{1}\left(x\right) & =\left({\bar{l}}_{U}-{\bar{l}}_{L}\right)\left\{1-f\left(x\right)\right\}f\left(x\right), \end{array}$$

(17) $$\begin{array}{cc} C_{2} & =\left[\begin{array}{ccc} 0 & \cdots & 0\\ -g_{2}\left(\gamma_{f2}\right)\sin\Psi_{2} & \; & 0\\ \vdots & \ddots & \\ -g_{2}\left(\gamma_{fn}\right)\sin\Psi_{n-1} & \cdots & -g_{2}\left(\gamma_{fn}\right)\sin\Psi_{n-1}\\ -g_{2}\left(\gamma_{b1}\right)\sin\Psi_{2} & \; & 0\\ \vdots & \ddots & \\ -g_{2}\left(\gamma_{bn-1}\right)\sin\Psi_{n-1} & \cdots & -g_{2}\left(\gamma_{bn-1}\right)\sin\Psi_{n-1} \end{array}\right], \end{array}$$

(18) $$\begin{array}{cc} g_{2}\left(x\right) & =\left({\bar{l}}_{U}-{\bar{l}}_{L}\right)f\left(x\right)+{\bar{l}}_{L}, \end{array}$$

and $\gamma_{f}={\left[\gamma_{f1},\cdots ,\gamma_{fn}\right]}^{⊤}$, $\gamma_{b}={\left[\gamma_{b1},\cdots ,\gamma_{bn-1}\right]}^{⊤}$, $\gamma_{all}={\left[\gamma_{f}^{⊤},\gamma_{b}^{⊤}\right]}^{⊤}$, $C_{1}\in R^{\left(2n-1)\times (2n-1\right)}$, $C_{2}\in R^{\left(2n-1)\times (n-1\right)}$.

By substituting Equation (14) into Equation (2), we can obtain the following:

(19) $$\begin{array}{cc} A\dot{w} & =B\bar{u}, \end{array}$$

(20) $$\begin{array}{cc} B & =\left[\begin{array}{ccc} B_{\phi } & B_{l}C_{1} & B_{l}C_{2} \end{array}\right], \end{array}$$

(21) $$\begin{array}{cc} \bar{u} & =\left[\begin{array}{c} \dot{\phi }\\ {\dot{\gamma }}_{all}\\ \dot{\psi } \end{array}\right]. \end{array}$$

The model (19) changes depending on the grounded/lifted status of each pair of wheels. When the *i*th wheel pair is not touching the ground, the *i*th row of (19) disappears. We allocated a unique index known as the *mode* to each model to represent the overall status of the wheel pair. When the mode is σ and the $i_{1}st,\cdots ,i_{n_{\sigma }}$ wheel does not touch the ground, Equation (19) becomes

(22) $$\begin{array}{cc} {\bar{A}}_{\sigma }\dot{w} & ={\bar{B}}_{\sigma }\bar{u}, \end{array}$$

(23) $$\begin{array}{cc} \; & =\left[{\bar{B}}_{\phi }\;\;{\bar{B}}_{l}C_{1}\;\;{\bar{B}}_{l}C_{2}\right]\bar{u}, \end{array}$$

where ${\bar{A}}_{\sigma }\in R^{(n-n_{\sigma })\times 3},{\bar{B}}_{\sigma }\in R^{\left(n-n_{\sigma }\right)\times \left(4n-2\right)}$, and ${\bar{B}}_{\phi }$ and ${\bar{B}}_{l}$ are the matrices in which the $i_{1},\cdots ,i_{n_{\sigma }}$th rows are eliminated from A and B, respectively.

In the following subsection, we consider the shift. The body area immediately behind the connecting part, which becomes the next connecting part when the shift is completed, is called the "connecting preparation part." In this method, the yaw joint angle of the connecting parts must be zero for the wheels to properly attach on the front plane, and the yaw joint angle of the connecting part and the connecting preparation part must be zero when shifting [10]. Therefore, the wheels behind the connecting part are ungrounded, and the yaw joint of the connecting preparation part is directly controlled to perform the shifting of the connecting part. The system also aims to reduce the influences of the shifting motion on the other parts of the connection by using the extension and shrinking of the links during the shift.

In mode σ, let ${\tilde{\phi }}_{\sigma }\in R^{n_{\phi \sigma }}$ be a vector of yaw joints that should be directly controlled in the connecting and connecting preparation parts, and $l_{\sigma }\in R^{n_{l\sigma }}$ be the link length that should be directly controlled, and ${\tilde{w}}_{\sigma }={\left[w^{⊤},l_{\sigma }^{⊤},\psi^{⊤},{\tilde{\phi }}_{\sigma }^{⊤}\right]}^{⊤}$. The kinematic model is obtained as

(24) $$\begin{array}{cc} {\tilde{A}}_{\sigma }{\dot{\tilde{w}}}_{\sigma } & ={\tilde{B}}_{\sigma }\bar{u}, \end{array}$$

(25) $$\begin{array}{cc} {\tilde{A}}_{\sigma } & =\left[\begin{array}{cccc} {\bar{A}}_{\sigma } & 0 & 0 & 0\\ 0 & I_{n_{l_{\sigma }}} & 0 & 0\\ 0 & 0 & I_{n-1} & 0\\ 0 & 0 & 0 & I_{n_{\phi_{\sigma }}} \end{array}\right], \end{array}$$

(26) $$\begin{array}{cc} {\tilde{B}}_{\sigma } & =\left[\begin{array}{ccc} {\bar{B}}_{\phi } & \bar{B_{l}}C_{1} & \bar{B_{l}}C_{2}\\ 0 & C_{1\sigma } & C_{2\sigma }\\ 0 & 0 & I_{n-1}\\ B_{\phi_{\sigma }}^{\prime } & 0 & 0 \end{array}\right], \end{array}$$

where $B_{\phi_{\sigma }}^{\prime }$ is a selection matrix whose components are zeros or ones, and satisfies $B_{\phi_{\sigma }}^{\prime }\bar{u}={\dot{\tilde{\phi }}}_{\sigma }$. $C_{1_{\sigma }},C_{2_{\sigma }}$ are matrices consisting only of the columns of $C_{1},C_{2}$ corresponding to $l_{\sigma }$, and $C_{1_{\sigma }}\in R^{n_{l\sigma }\times \left(2n-1\right)},C_{2_{\sigma }}\in R^{n_{l\sigma }\times \left(n-1\right)}$. Let us define $C_{\sigma }=\left[C_{1\sigma },C_{2\sigma }\right]$; then, $C_{\sigma }\bar{u}={\dot{l}}_{\sigma }$ is satisfied.

From (4) and (19), B is a row of full rank if $l_{fi}\neq 0$. Given that $l_{fi}>0$ in an actual robot, B is always a row of full rank. Given that $|\Psi_{i}|<\pi /2$, $\left[C_{1}\;\;C_{2}\right]$ is a row of full rank, $\left[C_{1\sigma }\;\;C_{2\sigma }\right]$ is also a row of full rank. Additionally, for ${\bar{B}}_{\phi }$ and $B_{\phi_{\sigma }}^{\prime }$, ${\left[{\bar{B}}_{\phi }^{⊤},\;B_{\phi_{\sigma }}^{\prime ⊤}\right]}^{⊤}$ have the property of being full rank. For example, if $n_{\sigma }=1$ and only the *i*th wheel does not touch the ground, then

(27) $$\left[\begin{array}{c} {\bar{B}}_{\phi }\\ B_{\phi_{\sigma }}^{\prime } \end{array}\right]=\left[\begin{array}{ccccccc} l_{f1} & 0 & \; & & \cdots & \; & 0\\ \; & \ddots & \; & & \; & & \vdots \\ \vdots & \; & l_{fi-1} & 0 & 0 & \cdots & 0\\ \; & \; & \cdots & * & l_{fi+1} & \; & 0\\ \; & \; & & \; & & \ddots & \\ {}* & \; & & \cdots & \; & & l_{fn}\\ 0 & \cdots & 0 & 1 & 0 & \cdots & 0 \end{array}\right].$$

If the wheel is not touching the ground, ${\bar{B}}_{\phi }$ is obtained by removing the row corresponding to the ungrounded wheel from $B_{\phi }$, but $B_{\phi_{\sigma }}^{\prime }$ is linearly independent of ${\bar{B}}_{\phi }$ as (27). Thus, (27) is always full rank.

This kinematic model is derived based on the velocity constraint of the wheel. Therefore, this model and the control method described below will not work for robots with isotropic friction characteristics (e.g., nonwheeled snake robots) or in environments with very low friction, because the error of the controlled variables increases owing to the error of the model.

## 3. Controller

**Figure 3:**
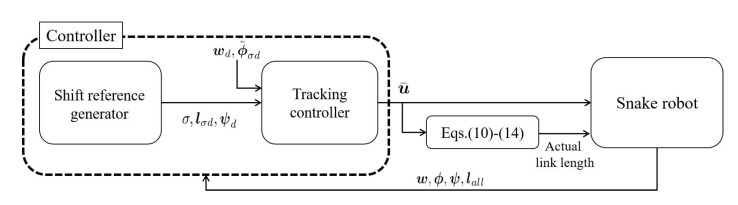
Controller.

The robot generates undulation motion by changing yaw joints and link lengths, and shifts the connecting part by changing pitch joints and link lengths. The control objectives are the tracking of the robot’s head to the target trajectory and moving between steps. These are the main tasks. In addition, the avoidance of singular configuration [23] is introduced as a secondary control objective (subtask). Figure 3 shows the controller configuration. The controller consists of a shift reference generator and a tracking controller. The shift reference generator determines the robot’s mode σ from the current robot’s situation, and calculates the target pitch joint angle $\phi_{d}$ and the target lengths of some links $l_{\sigma d}$ to realize step climbing. The trajectory tracking controller calculates the yaw joint rotational velocity $\dot{\phi }$ and the pitch joint rotational velocity $\dot{\psi }$, and mediating variable ${\dot{\gamma }}_{all}$ to achieve both tracking of the robot’s head to the target trajectory $w_{d}$ and the subtask. The ${\dot{\gamma }}_{all}$ is converted to an actual link length using (10)–(14), and is sent to the robot.

### 3.1. Trajectory Tracking Controller

For the kinematic model (24), the input $\bar{u}$ is designed as

(28) $$\begin{array}{cc} \bar{u} & =u_{T_{\sigma }}+u_{K_{\sigma }}, \end{array}$$

(29) $$\begin{array}{cc} u_{T\sigma } & ={\tilde{B}}_{\sigma }^{W†}{\tilde{A}}_{\sigma }\left\{{\dot{\tilde{w}}}_{\sigma d}-K_{\sigma }\left({\tilde{w}}_{\sigma }-{\tilde{w}}_{\sigma d}\right)\right\}, \end{array}$$

(30) $$\begin{array}{cc} u_{K\sigma } & =\left(I-{\tilde{B}}_{\sigma }^{W†}{\tilde{B}}_{\sigma }\right)K_{V}\eta , \end{array}$$

where ${\tilde{B}}_{\sigma }^{W†}$ is the weighted pseudoinverse of ${\tilde{B}}_{\sigma }$[24], $u_{T\sigma }$ is the input related to trajectory tracking, $u_{K\sigma }$ is the input related to redundancy, $K_{\sigma }$ is the positive definite diagonal matrix for gain, $K_{V}$ is the diagonal matrix for gain on redundancy, and η is an arbitrary vector. $u_{T\sigma }$ is the input for convergence of the control point to the target, and $u_{K\sigma }$ is the input related to the redundant component and does not interfere with $u_{T\sigma }$. Therefore, $u_{K\sigma }$ does not interfere with the convergence of the control point to the target. By properly designing the η, a secondary task can be accomplished simultaneously with the main task of trajectory tracking of the control point. The design of η is described below. As ${\tilde{B}}_{\sigma }$ is always full-rank, as explained in the previous section, from (19) and (28), we obtain the following closed loop system:

(31) $$\begin{array}{c} {\tilde{A}}_{\sigma }\left\{{\dot{w}}_{\sigma }-{\dot{w}}_{\sigma d}+K_{\sigma }\left(w_{\sigma }-w_{\sigma d}\right)\right\}=0. \end{array}$$

If ${\tilde{A}}_{\sigma }$ is a column of full rank, then $w_{\sigma }$ converges to $w_{\sigma d}$ as t→∞. However, the convergence of the controlled variable to the target value is not guaranteed when the column rank of ${\tilde{A}}_{\sigma }$ is not full. This case is called a singular configuration of the robot. The singular configurations resulting in rank deficiency of ${\tilde{A}}_{\sigma }$ are those in which all grounded wheel axes are parallel, or all extended lines of the axes with grounded wheels intersect at a single point [23]. In this study, these singular configurations are avoided by appropriately designing the input component related to redundancy $u_{K\sigma }$.

The $u_{K\sigma }$ in (28) is an input related to kinematic redundancy, which can achieve a subtask without affecting the trajectory tracking of the controlled variable. Let V(q) be the evaluation function and η be set as follows.

(32) $$\begin{array}{c} \eta ={\left[\frac{\partial V}{\partial \varphi_{1}},\cdots ,\frac{\partial V}{\partial \varphi_{4n-2}}\right]}^{⊤}, \end{array}$$

where $\varphi ={\left[\phi^{⊤},\gamma_{all}^{⊤},\psi^{⊤}\right]}^{⊤}\in R^{(4n-2)\times 1}$ and $\varphi_{i}$ is the *i*th element of φ. The time derivative of the evaluation function V(q) is derived as

(33) $$\begin{array}{cc} \frac{dV}{dt} & =\frac{\partial V}{\partial w}\dot{w}+\frac{\partial V}{\partial \varphi }\dot{\varphi } \end{array}$$

(34) $$\begin{array}{cc} \; & =\frac{\partial V}{\partial w}\dot{w}+\eta^{⊤}u_{T\sigma }+\eta^{⊤}u_{K\sigma } \end{array}$$

(35) $$\begin{array}{cc} \; & =\frac{\partial V}{\partial w}\dot{w}+\eta^{⊤}{\tilde{B}}_{\sigma }^{W†}{\tilde{A}}_{\sigma }\left\{{\dot{\tilde{w}}}_{\sigma d}-K_{\sigma }\left({\tilde{w}}_{\sigma }-{\tilde{w}}_{\sigma d}\right)\right\}+\eta^{⊤}\left(I-{\tilde{B}}_{\sigma }^{W†}{\tilde{B}}_{\sigma }\right)K_{V}\eta , \end{array}$$

where $(I-{\tilde{B}}_{\sigma }^{W†}{\tilde{B}}_{\sigma })\geq 0$. The η can contribute to increasing or decreasing *V* depending on the sign of $K_{V}$. The evaluation function *V* is designed as

(36) $$\begin{array}{cc} V & =a_{s}V_{s} \end{array}$$

(37) $$\begin{array}{cc} V_{s} & =\frac{1}{\det{\tilde{A}}_{\sigma }^{⊤}{\tilde{A}}_{\sigma }}, \end{array}$$

where $a_{s}$ is a positive constant representing the weights. $V_{s}$ is the evaluation function for singular configuration avoidance, which is *∞* when the configuration of the robot is singular. Singular configuration is avoided by decreasing *V* with negative values for each component of $K_{V}$.

### 3.2. Shift Reference Generator

The shift reference generator determines the robot’s mode σ based on the relative relationship between the robot, step, and the shift situation. If the shift start condition is satisfied, the shift of the connecting part is started, and the target values of the pitch joint angle and link length near the connecting part are calculated. The calculated target pitch joint angle $\psi_{d}$ and the target link length to be directly controlled $l_{\sigma d}$ are passed to the tracking controller, together with the mode σ.

**Figure 4:**
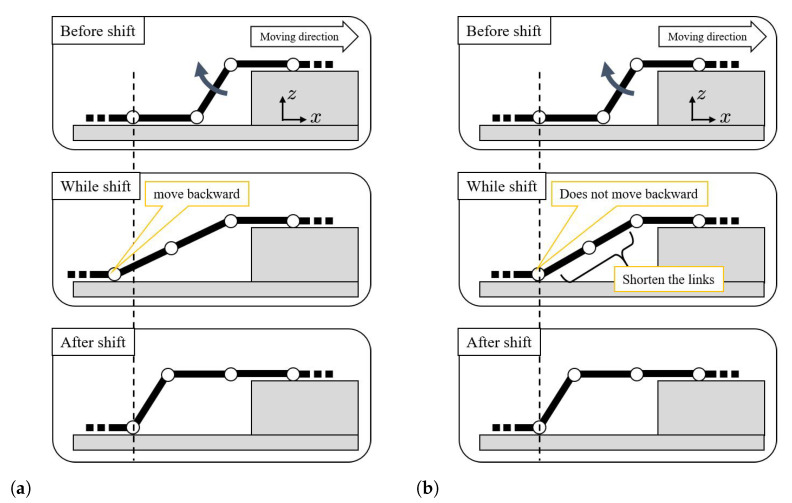
Shift motion in [10] and this paper. (**a**) Link length is fixed [10]. (**b**) Link length can be changed (this paper).

When shifting the connecting part, the motion is designed to make the relative distance between the two ends of the connecting part before and after the shift constant to minimize the effect of the shift on other parts of the trunk. The shift operation is shown in Figure 4. Note that all yaw joint angles were set to zero for simplicity for the figure, and the figure is viewed from the xz plane. Given that the link length was fixed in [10], as shown in Figure 4a, there was a risk in that the backward movement would occur during the shift, which would increase the joint angle of the body on the rear plane and cause it to exceed the limit value. In contrast, as the robot used in this study had changeable link lengths, the shift was performed so that there was no backward movement, as shown in Figure 4b. The specific target values of joint angles are omitted because they can be obtained only from plain geometrical relations.

The timing for starting the shift was set in the same way as [10]. Specifically, the shift was started when the yaw joint angle of the connecting part was near the target value (zero) and when the wheel ahead of the connecting part touched the ground.

## 4. Experiments

**Figure 5:**
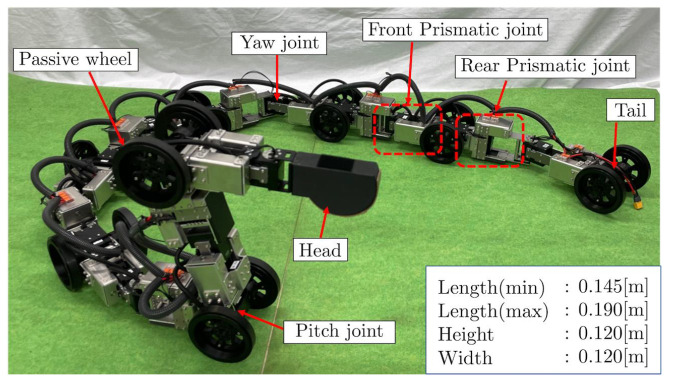
A snake robot with prismatic joints for experiments.

The robot developed for the demonstration of the effectiveness of the proposed control method is shown in Figure 5. This robot has yaw joints, pitch joints, passive wheels, and prismatic joint units. The link lengths can be changed using the prismatic joint units. In these units, a feed screw is rotated by the actuator through a flat gear, the screw nut translates along the feed screw, and the link length changes. Independent of the link length, the yaw joint can operate in the range of ±91 deg and the pitch joint in the range of ±110 deg.

This robot was developed for this study and can change the link lengths without interfering the motion range of its rotational joints. In addition, a link length limitation method [22] based on a sigmoidal function was verified for the first time using an actual robot.

The parameters used for the experiments are listed in Table 1. The experiment was conducted with motion capture equipment to obtain information on the head position and orientation. The robot climbed a step with a height of 0.20 m. When the robot was tested with a step height of h=0.20 m as a parameter in the input calculation, deformation occurred in the telescopic link owing to the insufficient rigidity of the robot; correspondingly, the robot could not move forward due to contact between the robot and the step. Therefore, we performed the experiment with h=0.23 m in the input calculation by taking into account the deformation of the robot.

As a result of prioritizing miniaturization and weight reduction, the prismatic joint unit had low-mechanical rigidity and was deformed when external forces acted on it. When a force was applied from the side, including during deformation, the frictional force generated inside the prismatic joint unit increased. However, the output of the unit was sufficiently large such that the telescopic motion was not inhibited, and the link length followed the target. The propulsion speed of the robot was reduced to satisfy the assumption that the wheels did not skid. At a low-propulsion speed (and a low acceleration), the force which acted on the wheel did not exceed the maximum static friction force, and the velocity constraint could be satisfied. As a result, the control error was small, and the robot could follow the target trajectory. In contrast, fast motion (high acceleration) caused the force acting on the wheel to exceed the maximum static friction force and increase the control errors due to wheel slippage. Therefore, this study slowed down the robot’s movement speed.

**Figure 6:**
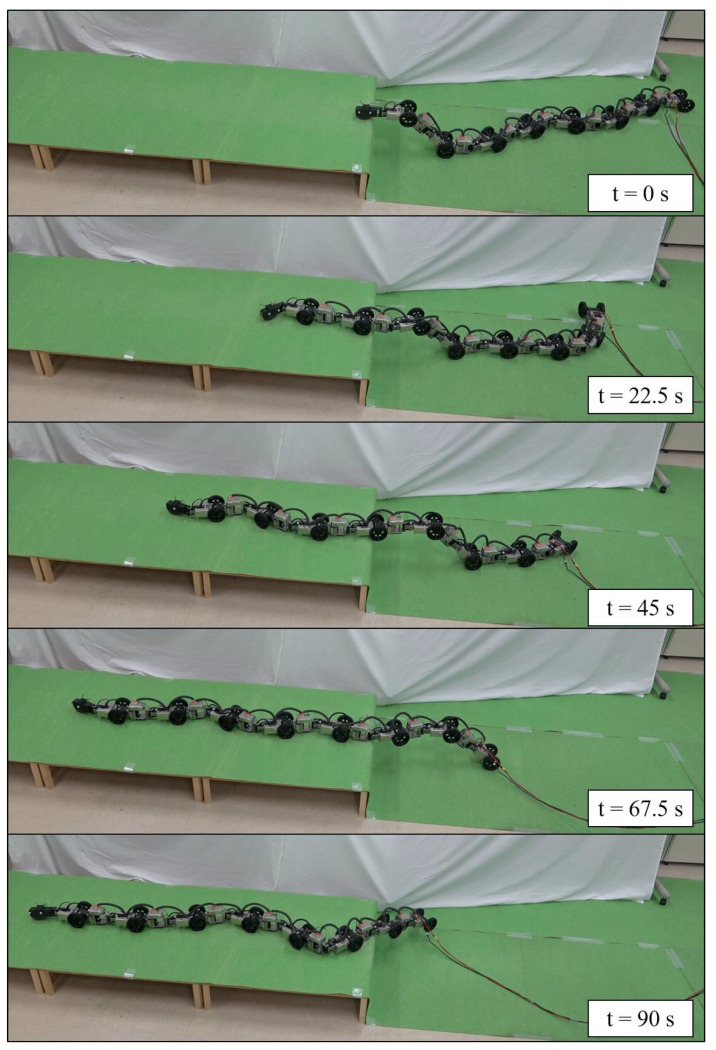
The robot motion in experiments.

**Figure 7:**
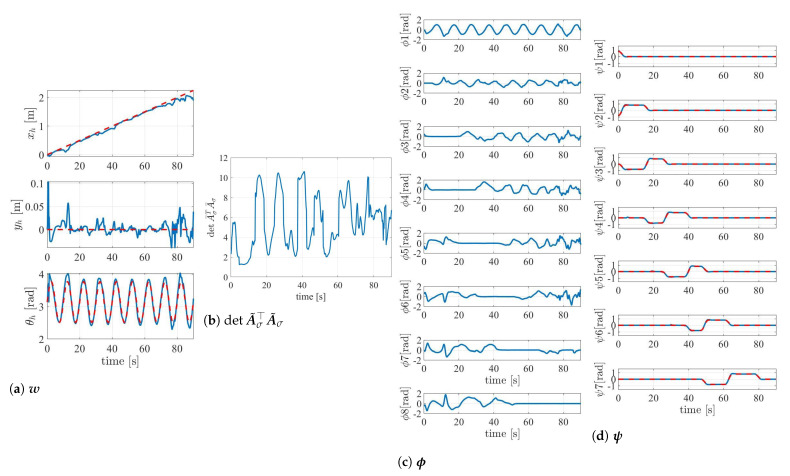
The results of w, det ${\tilde{A}}_{\sigma }^{⊤}{\tilde{A}}_{\sigma }$, ϕ, ψ.

**Figure 8:**
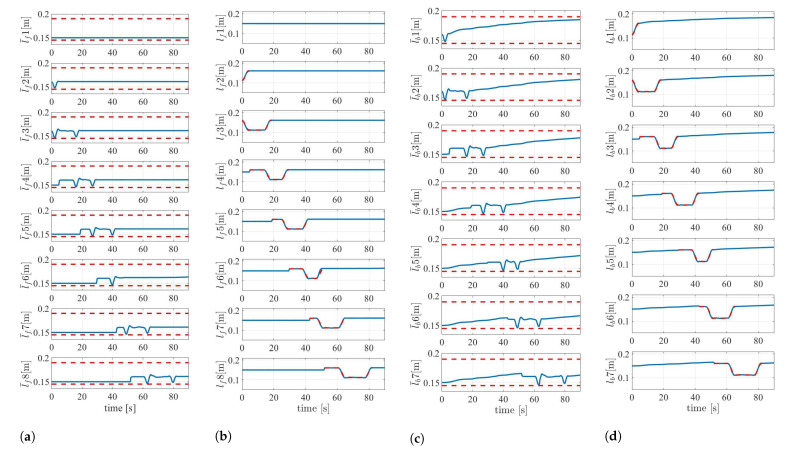
Experimental results of the link length. (**a**) The actual forward link length ${\bar{l}}_{f}$. (**b**) The forward link length on the projection model $l_{f}$. (**c**) The actual backward link length ${\bar{l}}_{b}$. (**d**) The backward link length on the projection model $l_{b}$.

Figure 6 shows the robot’s behavior during the experiment, and Figure 7 and Figure 8 show the results. The position and orientation of the head w tracked the target trajectory. In Figure 8b,d, the red-dotted line represents the target during the shift operation. It was confirmed that $l_{f}$ and $l_{b}$ also followed the target during the shift operation. With the exception of the periods during the shift operation, the link length was adaptively changed by the redundancy so that $l_{b}$ became longer. This is an effect of singular configuration avoidance by the redundancy similar to that reported in [22], and contributes to the amplification of the oscillations at the head toward the tail. The link lengths ${\bar{l}}_{f},{\bar{l}}_{b}$ were confirmed to be within the limit set by the sigmoidal function. The full rankness of ${\tilde{A}}_{\sigma }$ of the column depends on ${\bar{A}}_{\sigma }$. Thus, $\det{\bar{A}}_{\sigma }^{⊤}{\bar{A}}_{\sigma }$ was used for the evaluation of the singular configuration. If the robot is in a singular configuration, this value is set to zero. The determinant $\det{\bar{A}}_{\sigma }^{⊤}{\bar{A}}_{\sigma }$ did not converge to zero. This means that the singular configuration was avoided.

**Figure 9:**
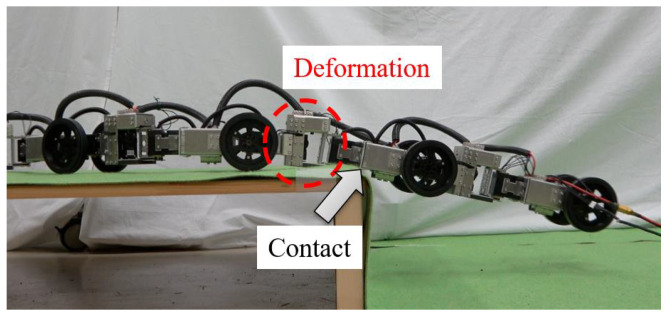
Contact between the robot and the step caused by deformation of the prismatic joint.

However, the error in the leading position configuration w increased after t=70. This was caused when the bottom part of the body contacted a step. As shown in Figure 9, the prismatic joint was deformed by the load of the tail lifted in a cantilever position after the tail shift, and the bottom part of the body contacted the step and could not be moved over the step to the tail. The same reason was applied to justify why it was necessary to use a larger value 0.23 m for the step height *h* set during control compared with the actual value 0.20 m. To solve this problem, it is necessary to improve the rigidity of the prismatic joints so that deformation does not occur. This is one of the subjects for future work.

## 5. Conclusions

We proposed a control method to realize step climbing for a snake-like robot with prismatic joints. The controller consisted of two parts: one was the shift reference generator which generated the joint movements for climbing the step. The other was the trajectory tracking controller which generated joint movements to track the head along the target trajectory. In this method, the prismatic joints were divided into those that were directly controlled for climbing steps and those that were represented as redundancies to perform continuous behavior. The standard sigmoidal function was used to indirectly represent the link lengths of the robot, and prevent the actual link lengths from exceeding their movable limits. A snake robot with prismatic joints was developed, and the effectiveness of the proposed method was demonstrated by experiments using the robot. Prevention of the backward movements of the trailing part by direct control of the link length and the shift motion of the connecting part, and prevention of exceeding the movable limit of the link length using the standard sigmoidal function, were confirmed.

Future studies will include the increase in the rigidity of the prismatic joints and the study of applications of the extensible degrees of freedom of the links in more complex environments.

## Figures and Tables

**Table 1:** Parameters of experiments.

Parameter	Value
*n*: The number of pairs of wheel	8
*l*: Head link length	0.100 m
$l_{f}\left(0\right)$: Initial value of $l_{f}$	${\left[0.150,0.112,0.161,0.150,0.150,0.150,0.150,0.150\right]}^{⊤}$ m
$l_{b}\left(0\right)$: Initial value of $l_{b}$	${\left[0.112,0.161,0.150,0.150,0.150,0.150,0.150\right]}^{⊤}$ m
ϕ(0): Initial value of ϕ	${\left[0,0,\pi /6,0,-\pi /6,\pi /6,0,-\pi /6\right]}^{⊤}$ deg
$l_{U}$: Maximum link length	0.190 m
$l_{L}$: Minimum link length	0.145 m
$W_{i}$: *i*th diagonal element of W	1.0(if i≤n), $1.0\times 10^{5}$(if n+1≤i)
Diagonal element of $K_{\sigma }$	The element corresponding to $y_{h}$, $l_{\sigma }$, others are 3, 2, 1, respectively.
Diagonal element of $K_{V}$	The element corresponding to $l_{fi}$, $l_{bi}$, others are −0.001, −2, −1, respectively.
$a_{s}$	$5.0\times 10^{-3}$
*h*: Height of the connecting part	0.23 m
$w_{d}$: Target trajectory of w	${\left[0.025t,0,\pi +\pi /5\sin\left(2\pi t/10\right)\right]}^{⊤}$
